# Alpha-Synuclein in Alcohol Use Disorder, Connections with Parkinson’s Disease and Potential Therapeutic Role of 5’ Untranslated Region-Directed Small Molecules

**DOI:** 10.3390/biom10101465

**Published:** 2020-10-21

**Authors:** Catherine M. Cahill, Rozaleen Aleyadeh, Jin Gao, Changning Wang, Jack T. Rogers

**Affiliations:** 1 Neurochemistry Laboratory, Department of Psychiatry, Massachusetts General Hospital and Harvard Medical School, Charlestown, MA 02129, USA; jack.rogers@mgh.harvard.edu; 2 Weill Cornell Medicine-Qatar, Qatar Foundation, Doha 24144, Qatar; roa2028@quatar-med.cornell.edu; 3 Department of Clinical Psychology, Qilu Hospital of Shandong University, Qingdao 266011, China; gaojin76@126.com; 4 Athinoula A. Martinos Center for Biomedical Imaging Massachusetts General Hospital and Harvard Medical School, Charlestown, MA 02129, USA; cwang15@mgh.harvard.edu

**Keywords:** alpha synuclein, alcohol, small molecules, iron homeostasis

## Abstract

Alpha-synuclein (α-Syn) is a 140-amino acid (aa) protein encoded by the Synuclein alpha SNCA gene. It is the synaptic protein associated with Parkinson’s disease (PD) and is the most highly expressed protein in the Lewy bodies associated with PD and other alpha synucleopathies, including Lewy body dementia (LBD) and multiple system atrophy (MSA). Iron deposits are present in the core of Lewy bodies, and there are reports suggesting that divalent metal ions including Cu^2+^ and Fe^2+^ enhance the aggregation of α-Syn. Differential expression of α-Syn is associated with alcohol use disorder (AUD), and specific genetic variants contribute to the risk for alcoholism, including alcohol craving. Spliced variants of α-Syn, leading to the expression of several shorter forms which are more prone to aggregation, are associated with both PD and AUD, and common transcript variants may be able to predict at-risk populations for some movement disorders or subtypes of PD, including secondary Parkinsonism. Both PD and AUD are associated with liver and brain iron dyshomeostasis. Research over the past decade has shown that α-Syn has iron import functions with an ability to oxidize the Fe^3+^ form of iron to Fe^2+^ to facilitate its entry into cells. Our prior research has identified an iron-responsive element (IRE) in the 5’ untranslated region (5’UTR) of α-Syn mRNA, and we have used the α-Syn 5’UTR to screen for small molecules that modulate its expression in the H4 neuronal cell line. These screens have led us to identify several interesting small molecules capable of both decreasing and increasing α-Syn expression and that may have the potential, together with the recently described mesenchymal stem cell therapies, to normalize α-Syn expression in different regions of the alcoholic and PD brain.

## 1. Introduction

Alpha-synuclein (α-Syn) was first discovered as a neuron-specific protein of 143 amino acids (aas) in purified cholinergic synaptic vesicles from the electric ray, *Torpedo californica*. Prior to its discovery, senile plaques of Alzheimer diseased (AD) brain were analyzed, leading to the identification of two unknown peptides in addition to amyloid beta (Aβ) named non-Aβ components of AD (NAC). Based on their hydrophobicity and their ability to form β-structures, they were thought to act as a “seed” for Aβ aggregation [1]. A 140-amino acid protein was then identified as the precursor of NAC (NACP) and named α-Syn and the smaller 134-amino acid protein was named β-synuclein (β-syn) [2,3]. It was later shown that α-Syn and β-Syn are expressed predominantly in the brain and are concentrated in the presynaptic terminals of nerve cells. Another synuclein expressed at high levels in ovarian and breast carcinomas was named γ-syn, and its’ gene was reported as breast cancer-specific gene 1 [4].

Alpha synuclein is thought to contribute to neurotransmission via the essential folding of SNARE proteins and the release of both dopamine (DA) and glutamate [5]. SNARE proteins mediate the fusion of vesicle laden neurotransmitters with their target membrane, and this is aided by α-Syn which promotes SNARE complex assembly [6]. Overexpression of wild-type α-Syn decreases DA neurotransmission by decreasing several important proteins: (1) tyrosine hydroxylase (TH) (the last enzyme involved in DA synthesis); (2) the vesicular monoamine transporter 2 (VMAT2), a membrane protein that transports neurotransmitters from the cytosol to the synaptic vesicles; and (3) the dopamine active transporter (DAT), which mediates DA uptake back into the cytosol from the synaptic cleft and increases DA efflux [7,8]. Therefore, long-term α-Syn overexpression decreases intra- and extra-cellular levels of DA, ultimately decreasing DA neurotransmission in the brain. In fact, DA by itself binds to the C-terminus of α-Syn and has been shown to reduce α-Syn aggregation. Therefore, compromised DA secretion (as occurs in the loss of DA neurons in the substantia nigra (SN) of PD patients) will lead to enhanced α-Syn misfolding [9]. Dynamic changes in α-Syn release at the presynaptic axon termini in the Ventral Tegmental Area (VTA) have recently been associated with a length of exposure to drugs of abuse, and they result in fine tuning of the complex afferent inputs to DA neurons, potentially altering DA output [10]. Alpha Syn levels were increased 3-fold in the DA neurons of the SN/VTA in chronic cocaine users, and this may put addicts at increased risk for developing motor abnormalities of PD [11]. Intracellular aggregates of α-Syn are the hallmarks of (PD), and several point mutations and genomic multiplications are linked to rare cases of familial PD [12]. In major depressive disorder (MDD), one of the prodromal features of PD, where a 30–60% comorbidity exists with all the alpha synucleopathies, is increased α-Syn expression in blood [13]. Alpha-Syn is also linked to Alcohol Use Disorder (AUD), and some genetic variants are linked to PD, AUD as well as associated disorders such as anxiety [14,15].

## 2. Alpha Synuclein Expression is Associated with Alcohol Use Disorder (AUD)

Genome-wide association studies (GWAS) have found α-Syn to be the topmost candidate gene for alcoholism, and single-nucleotide polymorphisms (SNPs) in intron 4 of the SNCA gene have been linked to alcohol craving [16,17]. Elevated blood alcohol of α-Syn protein and mRNA levels in human alcoholics are also associated with alcohol craving and dependence [16,18,19,20]. Increases in α-Syn in non-human primates consuming alcohol and in blood and brain regions of rodent models following alcohol administration or withdrawal have been demonstrated [19,21,22,23,24,25]. Alcohol withdrawal in a preclinical mouse model was associated with elevations in α-Syn protein levels in the amygdala, suggesting a role for long-term neuroadaptation from alcohol [24].

Microarray studies demonstrate alterations in α-Syn mRNA in the frontal and motor cortex of human alcoholics compared to controls [26,27,28,29]. Significant association of a genetic variant of SNCA (shorter REP1 variant) with reduced baseline expression in the prefrontal cortex (PFC), an area of the brain known to be affected by alcohol, has been reported [30]. The SNCA deficit hypothesis proposes that although low basal levels of SNCA in certain brain areas may predispose to cravings and enhanced neuronal activity, this then drives excessive alcohol consumption, leading to increases in SNCA [17]. In fact, α-Syn is downregulated in expression in the frontal cortex and caudate–putamen of inbred alcohol preferring rats [31], as well as in the brain (amygdala) and blood of the stress reactive DBP (D-Box binding protein) animal model of alcoholism prior to exposure to any alcohol. However, the opposite was found in the hippocampus in inbred alcohol-preferring rats when compared to non-preferring rats [32]. Rats bred for alcohol preference had higher levels of α-Syn and lower stimulated DA levels and more efficient DA re-uptake in the nucleus accumbens (NA) core than low drinking rats [33].Although other studies showed no differences in α-Syn levels in the amygdala and the NA, other areas of the brain, the cortex and striatum demonstrated low basal levels of α-Syn [31].

Variation in α-Syn levels in different brain regions may be attributed to α-Syn concentration and localization at the synapse, where it is predominantly found in DA neurons and where it promotes DA release. Degradation or specific proteolysis of α-Syn to shorter more aggregation-prone species may help explain the discrepant results, and the discovery of multiple α-Syn mRNA species that are likely to be prone to differential translational regulation likely leads to expression of specific isoforms in different brain areas in response to alcohol with varying abilities to modulate DA neurotransmission [18,19]. Because α-Syn binds to the DA transporter and increases the uptake of DA, modulation of α-Syn expression may affect the re-uptake of DA and alter neuronal signaling. DA signaling is thought to be the main inducer of craving in alcohol addiction. The DA reward pathways project to the dorsolateral prefrontal cortex (PFC) involved in executive functions which is altered in AUD. There are also alterations in GABAa receptors in the dorsolateral PFC of human alcoholics and the GABAa receptor and SNCA gene cluster in a region associated with alcohol abuse [34,35].

## 3. Parkinson’s Disease, Parkinsonism and Alcohol Use Disorder

Around 5–19% of patients have familial PD due to mendelian inheritance of genetic mutations, for instance, such as cases involving autosomal dominant mutations in the gene for α-Syn and other genes including Parkin and LRRK2. There are also common genetic variants which contribute to increased susceptibility, although α-Syn represents a convergence point [36,37] Nevertheless, the majority of patients have sporadic or idiopathic PD. Idiopathic Parkinson’s disease (IPD) is a multisystemic neurodegenerative disorder and is the dominant entity in the range of parkinsonian disorders among patients suffering from the typical locomotor symptoms of the disease [37,38]. Age at onset of familial PD is heterogeneous between individuals, and idiopathic PD usually manifests at 60 years of age or older [38]. A large number of other parkinsonism-type disorders have some or all of the clinical features of PD, and the clinical syndrome is referred to as “parkinsonism” [38]. Some parkinsonian disorders are chronic and progressive and are caused by an unknown process, for instance, exposure to various toxins or traumatic brain injury, while others have a clear genetic cause. Currently, the one feature that unifies parkinsonian disorders is nigrostriatal DA neuro degeneration. One of the most important features in secondary parkinsonism (SP) is non-responsiveness to DA replacement therapy and prior or ongoing exposure to neuroleptics. Drug-induced parkinsonism is known to be one of the most common reasons for SP [39].

At the epidemiological level, a history of AUD was shown to confer an increased risk of PD in both men and women with a higher risk at lower ages with PD [40]. Although low-to-moderate beer consumption had a lower PD risk, greater liquor consumption with higher alcohol content had a higher PD risk, while wine consumption did not have any associated risk of PD [41]. Beer contains a large amount of purine which may help raise plasma urate, a free radical scavenger linked to lower PD risk. Pure alcohol induces oxidative stress and acts as a pro-oxidant and may also be pro-inflammatory. Although alcohol has been suggested to protect from neurodegeneration and vascular disease, for example, flavonoids found in red wine might have neuro- and vascular-protective effects [42], other studies have shown a negative association between alcohol use and neurodegenerative diseases such as PD [41,43]. Discrepant results across studies are likely due to differences in study design, dietary assessment and confounders such as smoking and coffee consumption.

A wide variety of movement disorders are associated with alcoholism, including alcoholic tremor, asterixis, alcoholic cerebellar disorder and basal ganglia disorders, some of which could be classified within the clinical diagnosis of parkinsonism [44,45]. In addition, withdrawal from alcohol is sometimes complicated by transient basal ganglia dysfunction manifested by parkinsonism or chorea, although these syndromes are distinct from PD and are completely reversible [45,46]. It has been suggested that alcoholics with transient parkinsonism might have an underlying nigral degeneration and subclinical PD, and they may develop parkinsonian symptoms because of superimposed alcohol-induced disturbances [47,48]. However, it may be that the infrequent occurrence of alcohol abuse among PD patients may explain why a more direct association of PD with AUD may be under-recognized and may be a clinical subtype of PD not yet diagnosed [48]. Interestingly, a mouse model harboring the human PD-associated A30P α-Syn mutation showed increased motivation to alcohol associated with increases in CREB phosphorylation in several brain areas [25].

At the molecular level, alcohol is metabolized in the liver by ADH to the toxic acetaldehyde, which is then converted to acetic acid by alcohol dehydrogenase 1 (ALDH1) in the cytoplasm or ALDH2 in the mitochondria. Studies have shown that ALDH2 deficiency or excessive ADH activity leading to elevations in acetaldehyde can decrease excessive drinking. Currently, there is a paucity of research linking the major PD clinical entities of idiopathic PD and familial PD to AUD, and the major association of PD with alcoholism stems from the fact that some alleles of the ADH1 and ADH4 genes—for which there is a truncating G78 stop mutation (allele rs283413) and a functionally impaired allele comprising two linked SNPs (rs34925826 and rs11480228)—are associated with PD [49,50]. Aldehyde dehydrogenase 1 (ALDH1) is expressed strongly and selectively in the mesencephalic DA neurons, which may protect them against high intracellular levels of aldehydes, a byproduct of DA metabolism as well as alcohol. Notably, ALDH1 mRNA levels have been shown to be specifically downregulated in DA neurons in PD by the use of in situ hybridization. In fact ALDH, has a secondary function of converting dihydroxyphenylacetaldehyde (DOPAL), a toxic metabolite of DA to its corresponding acid, dihydroxyphenylacetic acid (DOPAC). ALDH is highly expressed in the corpus striatum likely due to the presence of large quantities of DA neurons [51]. In addition, DOPAL in the presence of divalent metal ions such as Cu^2+^ and Fe^2+^ induces the oligomerization of α-Syn, which may be associated with the fact that α-Syn has several binding sites for Cu^2+^ and two for Fe^2+^ [52,53]. In PD, there is evidence of inhibition of ALDH in residual DA terminals, resulting in accumulation of DOPAL [54] and ALDH1A1 being substantially decreased in the SN of PD patients, while mitochondrial ALDH2 is increased in the putamen [55]. Elevations in ALDH are expected to protect DA neurons from DOPAL induced α-Syn oligomerization [52,56]. Another molecular link between secondary parkinsonism provoked by alcohol is impairment of central DA mechanisms and DA receptor sensitivity [57] A better understanding of the association between ALDH, alcoholism and DA metabolism is warranted. Heavy drinking leads to elevated acetaldehyde levels, leading then to inhibited ALDH enzymes that subsequently cause these adverse effects [58]. The link between α-Syn protein and mRNA expression in secondary parkinsonism associated with alcohol abuse has yet to be explored.

## 4. Alcohol Affects the Cholinergic and Dopaminergic Systems in the Brain

High doses of alcohol can inhibit the release of the neurotransmitter ACh in the hippocampus, the brain’s key memory region. Acetylcholine is one of several important neurotransmitters involved in cognitive processes of learning and memory. Addiction results from the reinforcing properties of addictive drugs on brain reward systems, particularly on the mesolimbic dopamine (DA) system. Dopamine (DA) is a neurotransmitter that is associated with the reward centers of the brain, and alcohol enhances DA release. Dopamine (DA) neurons in the midbrain respond not only to rewards, reward-predicting cues and behavioral choices, but also to other variables such as distance to reward as well as movement [59]. It has been shown recently that subpopulations of DA neurons in the mouse brain are functionally clustered and transmit information about a subset of behavioral variables, as well as encoding rewards [59]. Prolonged alcohol use induces DA receptor super-sensitivity, which is an enhanced physiological, behavioral or biochemical response to DA agonists [57,58].

Dopaminergic cell bodies arise in the ventral tegmental area (VTA) of the brain and project to the nucleus accumbens (NA) and prefrontal cortex (PFC). The VTA–NA (i.e., the meso accumbal DA system) is an important part of the reward system, and DA mediates some of the reinforcing/addictive actions of alcohol [60]. Increased DA release in the NA is induced by alcohol and appears to play a role in addictive behaviors [61]. Acetylcholine (ACh) acting via the nicotinic acetylcholine receptors (nAchRs) in the VTA is also involved in the DA-enhancing effects of alcohol as well as nicotine. It is thought that ethanol can enhance agonist activation of the ACh channel by helping to maintain an open configuration [62,63]. It has been shown that alcohol increases VTA ACh release, and Ach then binds to the nAchR in the VTA, leading to increased DA in the NA [63]. Ethanol-induced excitation of DA neurons in the VTA can be attributed to inhibition of currents in M-channels [64]. However, a sub hypnotic dose of ethanol reduces DA turnover in the substantia nigra (SN) and caudate nucleus in the rat [65,66,67], and if these reductions in DA turnover in the SN are also seen in humans, it might help explain the manifestations of motor dysfunctions of parkinsonism-like features in some alcoholics during withdrawal [46,59,68,69].

## 5. Genomic Organization of the SNCA Gene and Association of the 115 SNCA Variant with Alcohol Use Disorder

**Figure 1:**
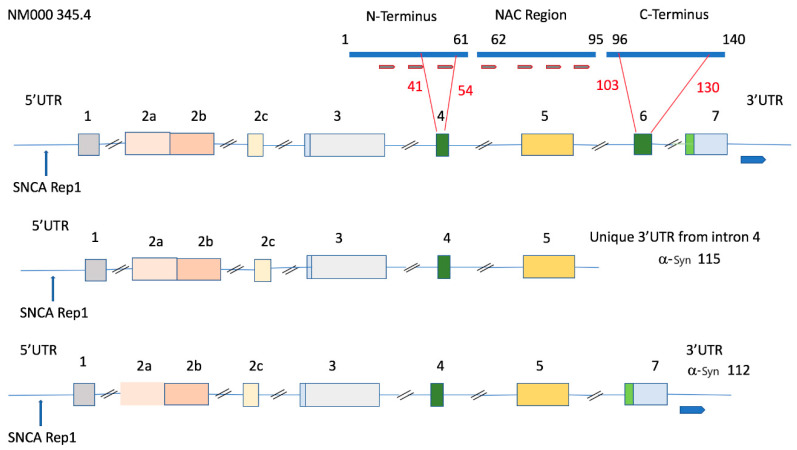
The main synuclein alpha (SNCA) transcript together with the shorter 115 and 112 variants associated with alpha-synuclein (α-Syn) protein aggregation. The NCBI accession number NM000345.4 is on the left. Exons are numbered with alternative 5’ untranslated region (5’UTR) exons (2a–c) and are colored light pink. The REP1 repeat sequence is located on the 5’UTR. The thick blue line (top) represents the full-length 140-amino acid protein derived from codifying exons 3–7. The short brown bars represent the seven imperfect KTKEGV repeats associated with tetramerization and protein aggregation. Transcripts lacking exon 6 lack the C-terminus, which prevents aggregation by masking these repeats. Exons 4 and 6 (dark green) are alternatively spliced exons giving rise to SNCA 112 (lacking exon 6), SNCA 126 (lacking exon 4) and SNCA 98 (lacking both exon 4 and 6) (not shown). Light blue indicates non-translated parts of exons 3 and 7, and light green indicates the corresponding SNCA-translated sequence. The SNCA 115 transcript associated with alcohol use disorder (AUD) encodes a 115-amino acid polypeptide coded from exons 3–5, whose transcript has a unique 3’UTR containing the first 393 nucleotides of intron 4.

The SNCA gene is around 117 kb in length and maps to chromosome 4q21.3-22 with other alcohol-associated genes, alcohol dehydrogenases ADH1B and ADH1C [70]. Until recently, the SNCA gene was believed to be a 6-exon gene (NCBI accession number: AF163864); however, novel GenBank data show that there are more (NCBI accession number: NG_011851). Of these exons, 1, 2a, 2b, and 2c are variably included in the 5’ untranslated region (5’UTR) in the different transcripts, and exons 3–7 are transcribed (Figure 1). Among the latter, exons 4 and 6 (corresponding to exons 3 and 5 in former GenBank data) are alternatively spliced and give rise to a minimum of four transcripts for each of the 5′UTR differing isoforms as follows: exon 3–7 containing transcripts, and transcripts lacking exon 6, exon 4, or exon 4 and 6. The different forms of each of these alternative transcripts differ primarily in their 5’ and 3’ regulatory regions, with the 3’UTR showing more variability in length, while 95% of the SNCA variants have the first 574 base pairs in common [70].

The full-length α-Syn protein encompasses three distinct regions: (1) an amphipathic NH terminal region that adopts an α-helical structure upon membrane binding; (2) a non-amyloid-β component (NAC) central region involved in protein aggregation; and (3) a highly acidic COOH-terminal region that masks the NAC region and reduces α-Syn aggregation (Figure 1). Alpha-Syn forms at least two types of aggregates: (i) non-toxic fibrillization-resistant tetramers observed under normal conditions and (ii) toxic dimers, trimers, and higher-order oligomers assembling into insoluble fibrils in disease 1. Bartels and colleagues have shown that endogenous α-Syn mainly occurs as a folded tetramer of about 58 kDa, which interacts by means of its central helices [71]. The N-terminal helices and the C-terminus do not directly interact with each other in their tetrameric state, and α-Syn does not undergo aggregation.

The wild-type 140-amino acid protein is the product of a gene containing seven exons [70]. The 126-amino acid variant is missing exon 4, and a newly identified 98-amino acid variant is missing both exon 4 and 6. The α-Syn 112 variant lacks exon 6 and shows deletion of the C-terminal amino acids 103–130 (Figure 1). In 2003, Murray and colleagues showed that C-terminal-truncated α-Syn proteins aggregate faster than the full-length molecule. The C-terminal negative charge influences the kinetics of α-Syn aggregation. The longer the C-terminal, the lower the aggregation rate of α-Syn. The α-Syn middle region constitutes the core of α-Syn filaments, and the C-terminal negative charges counteract α-Syn aggregation [72]. Different α-Syn variants are known to associate with disease. Reports show increased nigral SNCA-140 and SNCA-126 levels in PD patients [73]. The 126-amino acid mRNA variant was shown to be lower in dementia with Lewy bodies and AD. It has been suggested that the 126-aa variant with lower aggregation properties might be necessary for normal brain functioning during aging [74]. The brain specific 98-amino acid variant with N- and C-terminal deletions is reportedly overexpressed in the frontal cortices of patients with Lewy body dementia and AD. In fact, up to 15% of truncated α-Syn forms of varying length are present in Lewy body pathology. Generation of C-terminal truncations in α-Syn via several enzymatic cleavage sites demonstrated that these truncated species enhance α-Syn fibril assembly and promote the ability of full-length α-Syn to aggregate [70]. The α-Syn-112, whose C-terminal is shortened by more than 60%, significantly reduces the negatively charged amino acid content. The increased net charge of α-Syn-112 strongly enhances its aggregation properties. Although α-Syn-112 does not have the S129 phosphorylation site, it retains the secondary phosphorylation site at S87, which is important in the development of Lewy pathology [75].

The SNCA-115 transcript variant that includes exons 1–4 and the first 393 nucleotides of intron 4 encodes a 115-amino acid peptide. This variant is highly associated with alcoholism and has a completely unique 3’-UTR (Figure 1). The difference between the α-Syn 115 and α-Syn-112 transcript variants is the presence of the first 343 nucleotides of intron 4 in the 3’UTR and the absence of exon 7 in α-Syn-115. Therefore, this variant lacks the C-terminal amino acids necessary to prevent aggregation as well as a 3’UTR-containing intron 4, which has several SNPs associated with alcoholic traits [76]. The charge on this protein is five, which is higher than that of α-Syn-112 (charge of 1), making these 115 species highly aggregation prone. The full-length α-Syn 140 and truncated α-Syn 112 were significantly lower, while α-Syn-115 was higher in the brains of alcohol misusers with cirrhosis than in controls [76].

## 6. REP1 Alleles and Variations in the 5’ and 3’UTRs of α-Syn; Associations with Parkinson’s Disease and Alcohol Use Disorder

The SNCA gene is highly polymorphic. Variability in the length of a microsatellite repeat, known as REP1 and located approximately 10 kb upstream of the translation start site, is associated with differences in expression that have been reported to increase PD and AUD risk [77]. The association of REP1 alleles with PD has been reported to be independent to that of AUD [77]. This microsatellite repeat region binds to poly-(ADP-ribose) transferase/polymerase-1 (PARP-1), a nuclear DNA-binding protein, and when bound, it decreases the activity of the promoter. Six alleles have been identified, (263, 265, 267, 269, 271 and 273 bps) based on the size difference of two alleles each, with longer alleles associated with higher expression in neuroblastoma cell lines [78]. The 267-, 269-, and 271-bp alleles occurred the most frequently, although the frequency distribution varied among different ethnicities [79]. The 263 bp allele was more prevalent in PD cases compared to controls [18]. A more recent study observed population heterogeneity, where longer alleles increased PD risk, while some shorter alleles increased the risk of PD in Asian populations. Phenotypic analysis demonstrated that PD patients carrying the 271-bp allele were prone to early onset PD (while the 267-bp had the opposite effect [30].

Differences in expression, however, may be associated with the different tissues sampled, and levels of SNCA mRNA and protein in different brain regions may not reflect blood levels. One report detected a lower REP1 allele of the shorter type in postmortem prefrontal cortex derived from alcoholic patients, while another study demonstrated increases in the blood of the longer REP1 alleles of α-Syn expression during alcohol withdrawal and linked to craving [19,20]. This study demonstrated that longer alleles of REP1 led to a higher expression of α-Syn, which is associated with alcohol dependence, while the shorter alleles correlated with reduced expression of α-Syn in the dorsolateral prefrontal cortex of long-term alcoholics [30,80]. In fact, the drinking phase of alcoholism was associated with α-Syn promoter hyper-methylation and reduced expression [81].

Genetic variation in the 3’ and 5’ ends of the α-Syn gene are associated with variations in protein expression levels, with the 3’ end likely regulating RNA stability via interaction with specific RNA binding proteins and miRNAs [82,83]. We and others have examined the SNCA 5’UTR, a long and GC-rich region predicted to fold into a stable hairpin stem loop that is regulated by multiple signals, including iron [84,85]. Single-nucleotide polymorphisms (SNPs) in the SNCA gene are associated with alcohol dependence, and some SNPs in the SNCA gene have been associated with alcohol craving [18,86]. First, a haplotype block in the 3′UTR is more abundant in individuals who crave alcohol [16], and at least 8 SNPs associated with alcoholism were found in intron 4, one of which (rs356168) was also associated with PD [80] and shown to induce subtle increases in α-Syn mRNA using CRISPR/Cas9 editing in human pluripotent stem cells (HPSCs). Following differentiation into neural cells, it was shown that this non-coding region was likely the binding site of brain-specific transcription factors EMX2 and NKX6-1 [87].

## 7. Alpha Synuclein and Iron Homeostasis

There are no studies, to our knowledge, regarding the effects of iron dyshomeostasis of long-term alcohol abuse on neurodegenerative diseases such as PD or AD. Substantial damage to the substantia nigra, an area of the brain associated with PD, has been observed in chronic alcoholics, including necrosis, and increased melanin and iron deposition in the perivascular spaces [88]. Alcohol-induced brain damage occurs via multiple mechanisms and in multiple brain regions, including the hippocampus, cerebellum and limbic cortex, with varying effects depending on intoxication or withdrawal [89]. Some of the effects of alcohol include increased oxidative stress, increases in pro-inflammatory cytokines, reduction in synapses, glutamate excitotoxicity, and effects on neural stem cells and neural cell proliferation [90].

**Figure 2:**
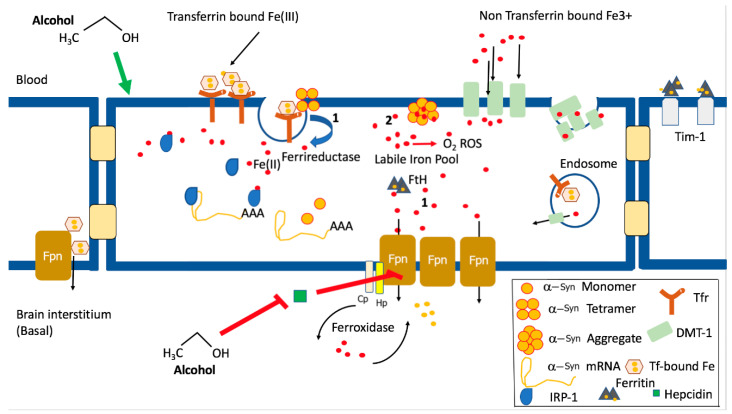
Uptake and export of iron from endothelial Cells of the blood–brain barrier are likely increased in alcohol use disorder, leading to brain iron dyshomeostasis. Alcohol misuse leads to systemic iron overload and to increased expression of α-synuclein. Recent evidence places apo- and holo-transferrin and Tim1 as central players in regulated iron loading at the blood–brain barrier (BBB). Ferritin is the primary storage form of iron in endothelial cells, and ferroportin is the main export pathway of iron across endothelial cells. (1) Alcohol reduces expression of the hormone hepcidin, leading to increases in the iron transport proteins DMT-1 and ferroportin, causing the build-up of the labile iron pool (LIP) Fe (II) and increased brain iron accumulation. (2) The mRNA of α-Syn has a uniquely configured iron responsive element (IRE) which binds to the translational repressor, iron regulatory protein 1 (IRP-1). Iron binding to the IRP removes it from the IRE, allowing the translation of α-Syn mRNA to proceed. (3) This leads to increases in α-Syn protein expression and increased functional α-Syn with intact ferriductase activity, leading to further increases in the LIP. (4) The resulting increase in α-Syn expression may lead to α-Syn aggregation and, in the absence of increased ferritin (FtH) for iron storage, increased oxidative stress and cell death.

Evidence suggests that mis-regulation of α-Syn mRNA translation plays a clear role in the pathology of PD, and this mechanism may also be important in AUD. Alcohol modulates α-Syn protein expression, and this may be via an iron-dependent mechanism. Increases in iron in the brains of alcoholics are likely to lead to differences in expression and aggregation of α-Syn (Figure 2). Alpha-synuclein itself has ferrireductase activity capable of reducing Fe^3+^ to Fe^2+^, which lends weight to the argument that its expression is likely regulated by the presence of iron [91,92]. An iron import function has been attributed to α-Syn, and its production of Fe^2+^ is important in making iron available as a co-factor for enzymes such as tyrosine hydroxylase, the enzyme involved in DA synthesis. Iron increases α-Syn aggregation, and iron chelation reduces insoluble α-Syn aggregates. In the α-Syn transgenic A53T mouse model of PD, an iron supplemented diet fed to neonates exacerbated motor and non-motor symptoms of PD. Iron levels are increased in neurons overexpressing α-Syn, and it has been shown that α-Syn binds both Fe^3+^ and Fe^2+^ and is involved in its cellular redistribution [93].

Iron homeostasis is achieved in part by post-transcriptional regulation through highly conserved messenger RNA (mRNA) stem loop motifs called iron response elements (IREs), which are present in the untranslated regions (UTRs) of a number of transcripts that are essential to iron metabolism. Iron response elements (IREs) are RNA stem loops in the UTRs of ferritin and transferrin-receptor mRNAs, where iron regulatory protein 1 and 2 (IRP1 and IRP2) bind to ferritin IREs to block their translation in Fe depletion and bind to TfR mRNAs and DMT-1 (iron import proteins) to stabilize their mRNAs [94,95]. In iron depletion, this mechanism enhances iron uptake into cells and stops it from being sequestered away for storage. IRP1 can therefore act as both a translational inhibitor and an enhancer to maintain iron homeostasis [96]. Our previous work reported a non canonical IRE in the 5’UTR of the Alzheimer’s disease (AD)-associated amyloid precursor protein (APP) 5’UTR. We used this target in high throughput screens (HTS) which led to the identification of several small molecules, including JTR-009 with APP and amyloid lowering efficacy [97,98]. We also identified a type 2 IRE in the 5’UTR of the α-Syn transcript, and this has been confirmed by others [84,85]. This IRE has been used as a therapeutic target for the identification of small molecules to specifically lower α-Syn expression [99]. Interestingly, although both βand γ-Syn show a high degree of homology to α-Syn at the protein sequence level, they do not contain IRE-like sequences within their respective mRNAs and do not prominently deposit with α-Syn in brain lesions, making the IRE on α-Syn a highly unique sequence and excellent therapeutic target. Our therapeutic strategy to limit α-Syn translation in PD and other synucleopathies by using our screened small molecules against the SNCA 5’UTR is ongoing.

## 8. Iron Dyshomeostasis and Alcohol Use Disorder

There is ample evidence that chronic alcohol abuse leads to abnormally high systemic iron (20–30% of alcohol abusers have systemic iron overload), which might also correlate with increased brain iron [100]. Even moderate drinking (2–3 drinks per day) is associated with increased indirect measures of iron stores as well as systemic iron overload and brain iron accumulation. Alcohol abuse is associated with uncontrolled intestinal iron absorption, increased liver iron and abnormal hepcidin signaling [100,101]. Hepcidin is an anti-microbial peptide hormone secreted from the liver whose secretion is decreased by alcohol. This leads to the increased expression of iron transport proteins, DMT-1 and ferroportin. Normally, hepcidin increases with iron overload and is decreased in iron deficiency, but alcohol renders hepcidin expression insensitive to iron overload [101]. Hepcidin binds to the iron exporter ferroportin and induces its internalization and degradation. Extracellular α-Syn and iron together lower hepcidin secretion from the liver, adding another layer of complexity in AUD and PD [102]. Liver failure can result in an increase in both body and brain iron deposition, and iron chelating agents have been used to protect against alcohol-induced oxidative brain damage [103]. The chemical properties of iron, although essential, are harmful, as they can catalyze the creation of reactive oxygen and nitrogen species via the Fenton reaction.

Iron is tightly controlled systemically, and is sequestered in vivo in ferritin for storage or transferrin for transport. The blood–brain barrier (BBB) prevents the passive transport of hydrophilic transferrin (Figure 2). The brain is dependent on the transferrin receptor-mediated transport of iron for its own local iron pool, separated from systemic iron fluctuations, and, more recently, the transport of iron-loaded ferritin shells via the Tim 1 receptor through endothelial cells of the blood–brain barrier has been demonstrated [104]. Iron distribution in the brain is variable but is primarily localized in deep grey matter basal ganglia where it can reach concentrations as high as those in the liver, the body’s main storage organ. With limited brain iron export, once non-heme iron enters the brain, it is thought to remain there. There are a wide range of brain disorders associated with iron dyshomeostasis, including AD, PD, multiple sclerosis (MS), amyotrophic lateral sclerosis (ALS) and others. Many are associated with excess iron accumulation, and several of these disorders share similar features to AUD, including motor and/or memory deficits, altered DA signaling and immune system deregulation.

Liver cirrhosis is associated with increases in serum Mn likely due to the inability of the liver to clear the toxin. Increased brain Mn is also associated with patients with hepatic encephalopathy (HE), and it is also associated with AUD and affects the globus pallidus, a part of the brain basal ganglia associated with involuntary movement which is also affected in PD. Increased amounts of Mn are deposited in astrocytes, causing Alzheimer’s type II changes and basal ganglia neuronal loss. The substantia nigra reticulata is also affected. Cirrhosis-related parkinsonism affects people with AUD, and this is due to manganese (Mn) deposition in the brain and is characterized by the usual PD motor symptoms, but it does not affect the nigrostriatal system [105,106,107,108]. Mechanistically, we have shown that exposure of DA neurons to Mn decreases the expression of the amyloid precursor protein (APP), an iron export protein associated with ferroportin, resulting in the accumulation of iron in these neurons and ferroptosis-mediated cell death [109]. Mn also induces α-Syn overexpression, which might also be associated with increases in iron import but nonetheless impairs synaptic vesicle fusion and therefore DA release [110]. It has also been shown that the Zn transporters ZIP8 and ZIP14 regulate Mn accumulation in brain endothelial cells which form the blood–brain barrier [111]. The role of these pathways in AUD is unknown.

It has been reported that gene expression patterns of both α-Syn and the transferrin receptor (TfR) are altered in the frontal and motor cortices of human alcoholics [26]. Interestingly, both TfRc and Aminolevulinic acid synthase (ALAS) involved in heme synthesis are upregulated in the liver of alcoholics, and TfRc (carbohydrate-deficient transferrin) has been established clinically as a biomarker of alcohol abuse, providing a reliable estimate of long-term alcohol intake [112,113,114]. The transferrin receptor and α-Syn have been shown to co-localize in retinal tissue, and their interaction is necessary for α-Syn effects on iron import [115,116]. Other reports of α-Syn ferrireductase activity support its role in cellular iron acquisition [92].

Ferritin-bound iron is the iron detected in quantitative susceptibility mapping (QSM), which can be used to measure iron content in deep grey matter and can detect altered deep grey matter iron both in aging and neurological disorders. A recent QSM study with data derived from fMRI from AUD patients has found a significant increase in iron in several deep brain grey matter structures, including the putamen/globus pallidus and red nucleus [117]. Reduced white matter volumes suggesting iron dyshomeostasis in alcoholics have also been demonstrated. The molecular mechanisms causing these white matter changes are yet to be elucidated [118,119]. Chronic ethanol exposure causes white matter (WM) atrophy and degeneration with major impairments in the structural integrity of myelin. The extent of WM lipid abnormalities suggests that ethanol broadly impairs molecular and biochemical functions regulating myelin synthesis, degradation, and maintenance in oligodendrocytes.

## 9. α-Synuclein 5’UTR-Directed Small Molecules as Potential Therapy for Alcohol Use Disorder and Parkinson’s Disease

The current medical treatments for alcohol are limited to only two FDA approved drugs: naltrexone and Antabuse. Naltrexone is an opiate antagonist which works by decreasing the craving for alcohol by producing an acute sensitivity. Antabuse works by inducing an aversion and sickness to alcohol by inhibiting acetaldehyde dehydrogenase (ALDH), preventing the oxidation of acetaldehyde to acetic acid. There is an urgent need for novel therapies for AUD that address biochemical mechanisms, and targeting α-Syn translation has the potential to offer a new approach to this disorder, which is one of the most prevalent addictions problems globally.

Alpha Syn translation is governed, at least in part, by an iron-responsive element (IRE) stem loop in the 5’ untranslated region (5’UTR) of its mRNA [84]. This stem-loop functions to confer iron-dependent post-transcriptional control [120]. Our laboratory first identified this 5’UTR IRE on α-Syn mRNA which was used in our previous screens to identify FDA-approved anti-α-Syn small molecules [84,121]. More recently, the approach of specific virtual screening and RNA targeting of the same IRE on the α-Syn 5’UTR has identified several specific molecules, synucleoids, which intercalate into the stem loop region of the unique SNCA IRE to modulate its translation [122]. These new small molecules targeting the 5’UTR of α-Syn mRNA together with our own novel panel of α-Syn 5’UTR-directed blockers described below, from our in vivo targeted screen, offer a much needed approach and new hope for anti-α-Syn therapy in PD and possibly AUD [99]. Our first early proof of principal studies employing a reporter construct containing the 5’UTR of SNCA which includes its unique 5’UTR IRE target, screened against a small 2000 FDA approved compound library, identified the cardiac glycosides, strophanthidine, digoxigenin and sarmentogenin, as well as the immunosuppressant mycophenolic-acetate, as 5’UTR-mediated SNCA translation inhibitors, exhibiting IC_50_s at less than 5 uM in a dose-response format with no toxicity [121]. Western blots later confirmed that strophanthidine reduced α-Syn expression in SK-N-SN/ SH-SY5Y, DA neurons, at an IC_50_ of <1 μM. Confirming specificity to SNCA 5’UTR mRNA, strophanthidine maintained β-actin and H-ferritin levels (IRE encoding control mRNA). In a follow up study, posiphen, a well-tolerated (+) enantiomer of phenserine (an anticholinesterase, AChE) and an APP 5’UTR-targeted small molecule (passing phase 1 clinical trials for AD), also blocked α-Syn translation via its 5’UTR both in neural SH-SY5 cells and in primary neurons from PAC-Tg SNCA mice expressing the human SNCA gene [123].

We then conducted a much larger high-throughput screen of 303,811 compounds from the NIH’s Molecular Library Probe Production Centers Network (MLPCN) library carried out at the Broad Institute in Cambridge MA using a similar reporter assay to screen for small molecules in order to identify more specific and potent compounds that could modulate SNCA translation [99]. Through this screening campaign, we have discovered a probe that specifically reduces α-Syn translation and likely acts by modulating an IRE/IRP 1 RNA–protein interaction. The probe, *N*,*N*-dimethyl-6-({[1-(1-naphthyl)-1*H*-tetrazol-5-yl]thio}methyl)-1,3,5-triazine-2,4-diamine, called Syn-516, inhibits IRE-driven α-Syn translation with an IC_50_ of 1.8 μM that has an over 100-fold selectivity, comparing cells transfected with a reporter construct lacking an IRE, (H4-C) or cells containing an IRE from the cellular prion, that is, IRE-containing 5’UTR (H4-PRP) [99]. Syn 516 is currently being tested in our in vivo and in vitro stem cell models of PD with very promising results, and it can also be tested in AUD models to mitigate alcohol craving associated with elevated α-Syn in blood and specific brain regions, notably the hippocampus and nucleus accumbens regions. We have also identified SNCA translation activators, including Syn 517, ML163, and *N*-naphthalen-1-yl-5-pyridin-4-yl-1,3,4-thiadiazol-2-amine, with an EC_50_ ≤ 10 μM and a greater than 10-fold selectivity as defined by EC_50_ ratios when comparing the primary screening cell line (H4-2a) to both the non-IRE-containing H4 cell line (H4-C) and the prion protein IRE-containing H4 cell line (H4-PRP). This compound could potentially be tested in animal models of AUD to investigate if increased protein levels of α-Syn could mitigate the executive dysfunction and addictive stage of AUD where there is a known association with low brain α-Syn expression specifically in the dorsolateral prefrontal cortex [124].

It will be important to first screen patients with AUD for the presence of SNCA IRE-containing transcripts. Where iron overload is a response to excess alcohol and iron increases SNCA expression via removal of IRP1 from the SNCA 5’UTR, targeting SNCA with our IRE-targeted SNCA blockers is a novel approach to treating AUD. Iron-responsive 5’UTR IRE-containing SNCA transcripts can potentially be targeted by our SNCA translation modulators to optimize SNCA expression when it is low (Syn-517) or to inhibit it when it is elevated (Syn-516). In short, identification of specific α-Syn transcripts with/without the IRE in biosamples derived from AUD and PD patients may shed greater light on the role of iron in the regulation of α-Syn in these diseases and help define clinical subtypes of diseases that would be more amenable to iron chelation therapy or small molecules directed to the α-Syn IRE to modulate its expression.

## 10. Stem Cells, α-Synuclein, and Alcohol Use Disorder

The role of impaired neurogenesis in neurodegenerative disorders such as PD and in AUD and the role of DA are areas of research requiring further attention. There is currently no effective therapies to prevent or slow down PD, and cell replacement therapy using human pluripotent stem cell (hPSC)-derived DA neurons holds considerable promise [125]. Several groups have derived clinical-grade mDA neuron precursors under clinical good manufacture practices which are progressing toward clinical testing in PD patients [126,127]. Mesenchymal stem cells are also showing promise in mouse and cellular models of PD where they have been shown capable of conferring neuronal protection by mediating the degradation of α-Syn oligomers [128,129]. A recent study reported that a single dose of human mesenchymal stem cells (MSCs) administered to rats bred to be high alcohol drinkers significantly reduced their voluntary alcohol intake [130,131]. Indeed, chronic use of alcohol is associated with an increase in neuroinflammation, which, by itself, can increase voluntary alcohol consumption. Reducing the size of MSCs by up to 75% using small spheroids allows them to penetrate the blood–brain barrier, resulting in reduced brain inflammation and oxidative stress in the animals that had consumed alcohol chronically [130]. Within 48 h of a single IV MSC treatment, the rats had reduced their alcohol intake by 90%. In addition, alcohol-induced neuroinflammation was significantly reduced, lasting 3 to 5 weeks following a single infusion. ^14^C dating analysis of postmortem human brains indicates that neural stem cells (NSCs) in the subependymal zone (SEZ) generate neurons for the striatum, a DA-producing area of the brain highly affected by alcohol [132,133]. A new role for α-Syn in the maintenance of NSCs in the SVC has been described, and this is likely due to its regulation of DA availability [134]. It has also been shown that α-Syn is necessary for the survival and differentiation of newly generated neurons in the dentate gyrus of the hippocampus and that DA depletion, together with α-Syn post-translational modifications, has an added detrimental effect [135].

## 11. Looking to the Future

There is a need to expand the number and type of AUD medications to include those that address one of its main side effects, i.e., iron overload and CNS neuroinflammation. Similar to PD, α-Syn overexpression and iron dyshomeostasis are hallmarks of this disease, and therapies that target α-Syn have potential to show new promise in AUD. Given SNCA’s role in cellular iron homeostasis, i.e., iron import in association with the transferrin receptor, its reported ferrireductase activity [136] as well as the occurrence of Iron Responsive Elements (IREs) on its regulatory 5’UTR region controlling its translation by iron [120], therapies that target SNCA may also be useful for the treatment of AUD, as well as related disorders such as anxiety and MDD, which have also been associated with iron dyshomeostasis [137,138]. Our screened molecules against the α-Syn Iron Response Element (IRE) containing 5’UTR, which, inhibiting α-Syn translation, may offer a much needed novel therapeutic approach to AUD, leading to a more specific targeted therapy as well as a deeper understanding of the biochemical mechanisms affected in the disease [120].
